# A Porcelain Inlay Ground to Fit a Cavity in a Tooth

**Published:** 1896-06

**Authors:** A. W. Harlan

**Affiliations:** Chicago, Ill.


### A Porcelain Inlay Ground to Fit a Cavity in a Tooth.

BY A. W. HARLAN, M.D., D.D.S., CHICAGO, ILL.

Read before the Mississippi Valley Dental Society, April 16, 1896.

I would not presume to call your attention to such a trivial subject if the whole question of inlays had not occupied such a large place in discussions before societies during the past six or seven years. Rubber inlays, platinum inlays, gold inlays, glass inlays, and baked porcelain inlays, with perhaps some others have had their advocates and do now I suppose in different quarters. The ground porcelain inlay, however, in labial cavities, buccal cavities and some crown cavities, to my mind furnishes us with something more artistic than any of the other methods of making or setting inlays. It is not a necessity to laboriously grind an inlay to fit the cavity in the tooth; this may be done with much greater expedition by preparing the cavity, taking an impression of it in plaster or modeling composition and getting a cast of the cavity, in copper amalgam, or Mellott's metal.

The inlay can be ground to fit the cavity to a hair's breadth, with diamond disks; undercuts and grooves may be made to provide for the flowing of the cement to retain the inlay in position. A tooth or bit of porcelain must be selected at least one shade darker than the moist natural tooth. If a porcelain rod is used it must be capable of retaining a high polish, or the difference in color will be too manifest. The edges of the inlay must not be beveled too much or the cement will show through them. The oxyphosphates are to be preferred for setting inlays in anterior teeth; gutta-percha or sulphur may be used for crown or buccal cavities in bicuspids or molars, or in pulpless teeth. The objection to gutta-percha is its color and the tendency to deterioration in such a small space as exists between an inlay and a cavity wall.

Canada balsam may be used, but it generally requires too much time and it is liable to become yellow from the secretions of the mouth. Nothing is more artistic than a section of a gum tooth with the gum attached to replace a waste spot on the neck of a superior cuspid or superior incisor.

A gold filling always looks badly and the thermal changes are so pronounced that the patient suffers every time the mouth is opened or a drink of water is taken, so that there can be no comparison as to the comfort of such an operation.

To more securely fix the labial inlays a small filling can be built over the edge of the inlay next the gum margins. This is done by grinding from the face of the inlay a depression of such shape that the gold can be adapted to the surface without disturbing the setting of the cement. Before setting an inlay with oxyphosphate, the cavity must be dried with ammonia fortior, and then with alcohol, and the inlay should be clean and dried in the same manner. After the inlay is set I allow at least half an hour before taking the rubber-dam from the tooth. A coat of copal ether varnish is used to protect it for a day or two. The reason I use porcelain in crown cavities in molars, is, that when the cavities can be made circular, it is easier to fill them; there are no thermal changes and it takes less than to use gold and it will probably last as long as gold.

DISCUSSION.

DR. L. E. CUSTER: The term inlay ordinarily applies to cavities in comparatively plane surfaces, such as the labial surface of an incisor or a crown cavity of a molar. Such a cavity has its margins still intact and of such shape that very little filling out above the cavity margins will be needed for restoration of contour. In such cavities, as we find them, the force of mastication comes either parallel to their surfaces or perpendicularly upon them. There is scarcely a position in the mouth where these inlays can be used but which will receive the force of mastication parallel or perpendicular to its surface. In crown cavities of molars this force is perpendicular to the surface of the inlay so that there is little danger of dislodgment. At this point I would call attention to the importance of making the inlay thick enough to withstand the force of mastication, and since porcelain is quite a good non-conductor of thermal changes, as stated in the paper, it may approach the pulp more nearly than a metallic filling. In incisors, however, the incisive force is parallel with the labial surface, so that if this force finds purchase upon the inlay there will be danger of dislodgment. Hence the importance of dressing the lower edge, at least flush with the enamel. Of course, where a gum inlay is inserted according to Dr. Thompson's method, the gum of the section must be flush with the gum. While this prominence above the root of the tooth at this point readily allows purchase of a downward force, this would only be accidental. The method of Dr. How should be used if the cavity can be made round without too much loss of enamel, because an inlay ground into a cavity is like a glass stopper, almost hermetically seals the cavity to begin with. In other cases, the process outlined by Dr. Harlan, I think, is the best method.

Before the impression is taken, there should be no undercuts, and it is scarcely necessary to make any afterward if the cavity is not too broad or shallow. Stiff modeling compound or gutta-percha would, I think, be preferable to plaster for this purpose. In cavities reaching to or under the gum, especially should the operator use this method of taking an impression, because it is very difficult to see the edge of the inlay at this point, but the stiff compound easily pushes the gum away in taking the impression.

The point that the inlay should be a shade darker can not be questioned. I think most inlays are too white, and this in thin ones is due, as stated, to the cement. Here is the importance of using a cement as near the tooth-color as possible.

I do not think anything equals cement for a setting material; sulphur and gutta-percha are open to the objection stated, and, besides, have no advantages but which are met as well with cement.

If the cervical border of an inlay is to be protected by a line of gold, this part of the cavity should be prepared with that end in view at the beginning, and care will be necessary in the insertion of the gold. There is another advantage in using gold at this point. We all know that cement is not to be trusted at this point, and the operator will also be more likely to get a better result by filling this part with gold, for the reason that he can see better to joint the inlay.

I object to the use of ammonia as a final wash for such or any cavity which is to receive cement, for the reason that ammonia is the best solvent for cement that we have. It is well to leave the inlay higher than the enamel until the cement has entirely set, and dress at a subsequent sitting. In polishing the ground surface I would recommend the use of a rubber wheel made by Johnson & Lund; it has a material incorporated in it which not only cuts porcelain, but polishes as well. They are only intended for lathe work, but can readily be cut into small wheels and mounted for the engine.

Dr. Harlan: In addition to the specimens of root-filling I here show you, some fifty or sixty operators in Chicago were requested to fill some root canals, using what material they thought best. From these I have found that salol is the poorest material for the purpose and oxychloride of zinc next.

